# Associations of Maternal-Infant Bonding with Maternal Mental Health, Infant’s Characteristics and Socio-Demographical Variables in the Early Postpartum Period: A Cross-Sectional Study

**DOI:** 10.3390/ijerph18168517

**Published:** 2021-08-12

**Authors:** Łucja Bieleninik, Karolina Lutkiewicz, Mariusz Cieślak, Joanna Preis-Orlikowska, Mariola Bidzan

**Affiliations:** 1 Department of Clinical and Health Psychology, Faculty of Social Sciences, Institute of Psychology, University of Gdańsk, 80-309 Gdańsk, Poland; karolina.lutkiewicz@ug.edu.pl (K.L.); mariola.bidzan@ug.edu.pl (M.B.); 2 GAMUT—The Grieg Academy Music Therapy Research Centre, NORCE Norwegian Research Centre, 5838 Bergen, Norway; 3 Faculty of Educational Sciences, Institute of Psychology, University of Lodz, 91-433 Lodz, Poland; mariusz.cieslak@now.uni.lodz.pl; 4 Division of Obstetrics, Division of Neonatology, Department of Perinatology, Faculty of Medicine, Medical University of Gdańsk, 80-214 Gdańsk, Poland; jpreis@gumed.edu.pl

**Keywords:** prematurity, NICU, maternal bonding, parenting stress, postpartum depression, anxiety

## Abstract

(1) Background: There is a continuing discussion concerning the impact of preterm birth on Maternal-Infant bonding with inconsistent results. The large burden of preterm births calls for research to evaluate the impact of it on material psychological outcome in the early postpartum period. Thus, the aim of this study was to evaluate the relationship between maternal postpartum bonding with maternal mental health, socio-demographical factors, and child’s characteristics. (2) Methods: A cross-sectional study design was used. In total, 72 women (a mean age of 31.44 years old) of preterm infants (mean gestational age = 33.54; range 24–36) filled out socio-demographic questionnaires, Postpartum Bonding Questionnaire (PBQ), Edinburgh Postpartum Depression Scale (EPDS), Postpartum Depression Screening Scale (PDSS), Generalized Anxiety Disorder Assessment (GAD-7), and Parental Stress Scale (PSS) 1–3 days post-delivery; (3) Results: The results analyses have shown positive correlations between the overall result of maternal postpartum bonding with stress (*p* < 0.01), maternal educational level (*p* < 0.01), maternal age (*p* < 0.05) and the number of children (*p* < 0.01). However, there were no significant relationships between other investigated variables. The results of linear regression have revelated the important role of the overall scores in experience of stress among mothers (explaining 49% of the variability). The mediating role of maternal stress on maternal postpartum bonding was not found. That relationship of maternal postpartum bonding and maternal stress was not moderated through socio-demographic variables. (4) Conclusions: In this study mothers of prematurely born children had a good level of Maternal-Infant bonding. Maternal stress was found to be a predictor of maternal postpartum bonding among the tested variables. Surprisingly, the study results did not show significant relationships between maternal postpartum bonding and maternal mental health (depression and anxiety).

## 1. Introduction

### 1.1. Medical Aspects of Preterm Birth

Preterm birth (PTB) is defined as childbirth prior to 37 weeks of pregnancy [1]. PTB is one of the most complex issues related to pregnancy due to its etiology, high frequency, high neonatal morbidity, mortality, comorbidity as well as short and long-term consequences. Spontaneous preterm birth (first subtype) is related to a mother’s age at pregnancy, pregnancy spacing, multiple pregnancy, infection, underlying maternal chronic medical conditions, nutritional, lifestyle, work, maternal psychological health, and genetic factors, among others. Provider-initiated preterm birth (second subtype) is linked with medical induction or cesarean birth due to obstetric indication, fetal indication, or other medically indicated factors [2]. According to The Global Action Report on Preterm Birth published by the World Health Organization based on research conducted in 148 countries (including Poland), approximately 15 million babies are born prematurely each year, corresponding to a 11.1% prematurity rate worldwide [3]. Regional analysis showed that the proportion of global PTB was the highest in Asia (52.9%) and sub-Saharan Africa (28.2%), followed by 7.2% in Latin America and the Caribbean, 5.2% in North Africa, 4.7% in Europe, and 3.2% in North America. From data collected in 107 countries, the highest rate of preterm birth was observed in Bangladesh (19.1%), Tanzania (16.6%), and India (13.6%) [4]. The number of PTB in Poland estimates at 6.7% [5]. 

Prematurity may lead to adverse effects on survival as well as medical, physiological, neurophysiological, and intellectual outcomes of a new-born child. Preterm neonates are at increased risk of death from the first 4 weeks of life up to 5 years of age [6]. They have increased morbidities in the postnatal period (e.g., respiratory distress syndrome, retinopathy of prematurity, infection, sepsis, and feeding difficulties) as well as adverse specific physical outcomes and neurodevelopmental/behavioral disorders over their lifespan [3,7]. Preterm birth is one of the major contributors to long-term adverse impacts on family (psychosocial, emotional, and economic), health services (costs of care), and intergenerational consequences (risk of preterm birth in offspring) [3]. The economic burden linked with prematurity includes a high cost of neonatal care, high long-term costs associated with complex health status, as well as the lost economic productivity over the life span [8]. Gestational age is one of most important predictors of mortality and morbidity [9], in particular, newborns with lower gestational age cut-offs are far more likely to have adverse neurodevelopmental outcomes [10,11]. There are four sub-categories of preterm birth with regard to gestational age at delivery: extremely preterm (<28 weeks), very preterm (28 to 31 weeks and 6 days), moderate preterm (32 to 33 weeks and 6 days), and late preterm (34 to 36 weeks and 6 days) [12]. Research by Manuck et al. [9] has shown that there is a pattern which indicates that with each additional week of gestation, the child’s survival rate increases and, at the same time, the length of initial hospitalization decreases. 

### 1.2. Psychological Aspects of Preterm Birth

Undeniably, premature delivery of a baby it is an emotionally difficult and stressful event not only for the child and their parents/caregivers but also for relationships in the whole family unit [13,14]. PTB is perceived by parents as a traumatic event [15] that disturbs the transition to parenthood. Mothers who experienced PTB and have their new-born hospitalized in the NICU are vulnerable to a long-lasting postpartum posttraumatic stress disorder (PTSD) [16,17,18] and postpartum depression in assessments performed up to 6 months after childbirth [19]. Grekin and O’Hara [20], based on a meta-analysis, have reported that the mean prevalence of postpartum PTSD was oscillated at 3.1% with the following risk factors: psychiatric history, postpartum depression, and complications during pregnancy, labor, and delivery. The authors also found an association between gestational age at delivery and the risk of having maternal posttraumatic symptoms, in which the earlier the pregnancy was terminated, the greater the risk of developing the disorder [20]. In terms of postpartum depression among mothers of premature infants, Vigod with colleagues [21] provided a systematic review which revealed that postpartum depression rates were around 40% in the early postpartum period, while earlier gestational age, lower birth weight, ongoing infant illness/disability, and perceived lack of social support were risk factors of sustained depression. The most current data indicates that the prevalence of postpartum depression among mothers of preterm infants varies from 6.6%, to 42.9% [19]. Various emotions such as worrying and fearing about a baby’s health conditions, unstable health situation of a child, and high risk of neurodevelopmental delay and childhood disability have altogether a strong impact on the level of parental stress and anxiety. Previous research has shown that mothers of prematurely born children experience significantly higher levels of stress in comparison with mothers of full-term babies [14,22]. Mothers after PTB experienced high levels of stress, while the most significant source of it concerned alterations to the parental role influenced by significant factors such as distance from the hospital and marital status [23]. According to the parenting stress theory, there are three groups of stressors: those associated with the parents such as parental sense of competence and the attachment with a child, those associated with a child such as mood or adaptation to the situation, and social stressors such as stressful life events [24]. Studies have shown that PTB can trigger other factors that can increase parental stress. For example, separation from the newborn baby due to the admission to the NICU and prolonged hospitalization not only causes additional parental stress and anxiety but also negatively impacts the sense of a parental role [25], including a lack of competence and skills in caring for a child [26]. PTB can also impact the presence of distress symptoms [14,27]. Research by DeMier with colleagues [28] has revelated that infant maturity and complications play a role as significant predictors of postnatal emotional distress in mothers. 

Maternal-Infant bonding is a concept described as an emotional tie between the mother and her baby [29]. The beginning of the bonding process can occur already during pregnancy and continue to grow after the child’s birth [30]. It is reported that disruptions in the maternal bonding process appear in 6–41% of dyads between a mother and her baby [31]. There are many factors that can impact the process of building emotional connections with the baby such as preterm birth [32]. Maternal-Infant bonding is even more significant in the experience of premature birth due to the infant’s perinatal risk status, long-term outcomes for the child’s development, parental mental health, and restrictions in NICUs, as parents do not have a similar opportunity to foster these connections with the premature baby in comparison to parents of full-term babies. Limited parental presence and interactions such as skin-to-skin contact may also disturb the process of building a parental relationship with the newborn baby [32].

There is a continuing discussion concerning the impact of PTB on Maternal-Infant bonding for the last few decades. However, there is a limited number of studies investigating the PTB impact in the early postpartum period [14,33]. One of the current studies by Trumello and colleagues [34] has suggested that PTB and its consequences (such as the child’s hospitalization) might negatively impact the mothers’ emotional state and her perception of her parental self-image, and through this, experience an adverse impact on the early bonding with the child. A small study that included 10 mothers from Tunisia has shown that the psychological state of mothers who experienced PTB has an impact on postanal bonding, while depression and anxiety adversely influence bonding after delivery [35]. Results obtained by Hall and colleagues [36] showed that, based on gestational age, mothers after PTB presented higher bonding feelings in comparison with mothers of full-term babies. However, not only medical conditions or parental interactions with the baby may influence bonding processes. The evidence suggests that the severity of the health status of the infant [37] and visible infant characteristics connected with prematurity also have effects on the bonding process with the newborn child [38]. Hoffenkamp et al. [39] demonstrated that preterm infants are less physically attractive in their parents’ perceptions in comparison with full-terms babies. In another study, low birth weight, which is connected to the babies’ appearance, was judged as less cute and less attractive than full-term babies [40].

### 1.3. Objectives

The Preterm Birth Priority Setting Partnership in the UK established the top 15 research priorities for preterm birth where bonding played one of the key roles (Priority research number 9: “What emotional and practical support improves attachment and bonding, and does the provision of such support improve outcomes for premature babies and their families?”) [41]. As the experiences of mothers after PTB in the early post-partum period is not fully established in the literature, the aim of this study was to explore maternal postpartum bonding and its relationship with a mother’s mental health, socio-demographical factors, and a child’s characteristics immediately after premature birth. 

This paper reports on a study that evaluated the following:

1. What is the maternal postpartum bonding score among mothers after a preterm birth?
2. What are scores of stress, anxiety, and depressive symptoms among mothers after a preterm birth?
3. Is postpartum bonding associated with maternal mental health (depressive symptoms, anxiety, and stress)?
4. Is postpartum bonding associated with a child’s characteristics (gestational age, birth weight, and final APGAR score)?
5. Is postpartum bonding associated with socio-demographical variables (age, education level, and number of children in the household)?

## 2. Materials and Methods

### 2.1. Study Design and Procedures

The results of this paper present a dataset of a larger longitudinal study concerning the impact of premature childbirth on parental outcomes during the baby’s NICU stay and beyond (ClinicalTrials.gov ID: NCT04118751). Ethics approval was obtained from the Research Ethics Board at the University of Gdansk (number 7/2019, date of approval: 29 April 2019). Individuals were enrolled at the Neonatology, Gynecology, and Obstetrics Ward of the Medical University of Gdańsk (Poland) by trained assistants who identified mothers who experienced preterm delivery through medical records. Trained assistants were responsible for the first contact with potential participants in the early postpartum period (1–3 days after childbirth) in order to provide them oral and written information about the project, alongside an informed consent form to sign. Participation in the project was voluntary. Each participant had the right to refuse to participate in the study or to withdraw from it at any time for any reason. It is important to mention that in Poland, there is a hospital policy according to which mothers after childbirth stay for 3 days in a hospital; thus, we conducted our study 1–3 days after delivery. Criteria for selecting the subjects were as follows:

Inclusion Criteria:

Primary inclusion criteria for the participants included:

- Woman in the reproductive age between 18 and 49 years and 11 months. The motivation for the cut-off age limit was due to the role of age in reproductive health (e.g., mothers higher in age are vulnerable to infertility, fetal anomalies, pregnancy loss, obstetric complications, and stillbirth) and mental health (e.g., lower age is related to depression, substance abuse, and posttraumatic stress disorder) [42,43,44,45].
- Parents of children born below the 37th week of pregnancy (clinical group) or above the 37th week of pregnancy (control group),
- Those that agreed to participate in the study after having completed information disclosures assessments and gave consent.

Exclusion Criteria:

Parents could not participate in the study if one of them suffered from a serious mental illness due to its potential impact on outcomes. 

For the purpose of this paper, only datasets of mothers who gave premature birth were included, while results of fathers and parents of full-term babies were already published separately (respectively: [46,47]).

### 2.2. Variables and Measurement Tools

Individuals were asked to complete anonymous paper datasets at their convenience within 3 days after birth of their child. 

#### 2.2.1. Maternal Postpartum Bonding

Maternal postpartum bonding was measured using the Postpartum Bonding Questionnaire (PBQ, [48]; Polish translation for Bieleninik (Dr Łucja Bieleninik obtained the written consent of the author of the questionnaire to use the author’s translated version [private correspondence with Professor Ian Brockington, 27 November 2018])). PBQ is a self-report questionnaire consisting of 25 items evaluated on a 6-point Likert scale (each 0–5, with the scale points labelled “always”, “very often”, “quite often”, “sometimes”, “rarely”, and “never”) to assess disorders in mother–infant relationships. The sum of points ranges from 0 to 125, with higher scores indicating problematic bonding. The questionnaire is divided into 4 scales (factors): a general factor (factor 1 indicating impaired bonding); rejection and pathological anger (factor 2 indicating mothers with severe disorders); anxiety about the infant (factor 3 showing anxiety about the care of the baby in anxious mothers); and incipient abuse (factor 4 signaling the risk of abuse requiring urgent intervention). Inter-rater reliability coefficients were satisfactory for factor 1, 2, and 3, with an exception for factor 4 (respectively: 0.95, 0.95, 0.93, and 0.77) [48]. The questionnaire is used in clinical practice and research worldwide; however, due to the lack of Polish adaptation and an inconsistency in the reliability and validity of factor 3 and 4 [49,50,51], we first used an exploratory factor analysis (EFA) to uncover the underlying structure of PBQ between items based on the included population. We employed principal component analysis with a varimax rotation in EFA. Reliability was calculated using Cronbach’s alpha coefficient to determine all internal consistencies of the scale (95% confidence interval). The suitability of data for EFA was calculated by Bartlett’s test of sphericity, χ^2^(276) = 774.54 *p* < 0.001, and the Kaiser–Meyer–Olkin measure of sampling adequacy (0.61). We used the Kaiser’s criterion (eigenvalues above 1) to obtain the number of factors to be retained and ensured consideration for the amount of variance with the factor solution. In addition, the two criteria favored the four-factor structure (49% of the total variance explained using 22 items after excluding those with a factor loading of less than 0.3). We found that the assignment of items to factors differed in comparison to the original version of the tool (see Reference [49]). Based on the obtained EFA results, we had decided that in further analyses, we would use only the overall scale score that is calculated as the sum of 22 PBQ items without factorability. The Cronbach α coefficient of reliability in this study group was 0.80 for the full scale.

#### 2.2.2. Maternal Postpartum Depressive Symptoms

Maternal postpartum depressive symptoms were measured using the Edinburgh Postpartum Depression Scale (EPDS [52], Polish translation [53]). This self-report scale consists of 10 items evaluated on a 4-point Likert scale (each 0–3) used as a screening tool to assess the symptoms of postpartum depression in mothers. The sum of the results range from 0 to 30, with high results indicating more severe depressive symptoms [52]. Due to its good psychometric properties, EPDS is a measure used around the world [54]. The Cronbach α coefficients of reliability in the Polish version of EPDS were calculated as 0.91. Intraclass correlation equaled to 0.95 with the scales’ sensitivity of 96% and the specificity was calculated as 93% for the cut-off of 13 points [55]. EPDS is well accepted by Polish mothers as a measurement tool for screening for postpartum depression [56]. In this study, the Cronbach’s alpha reliability coefficient was 0.83 for the full scale.

#### 2.2.3. Maternal Anxiety

Maternal anxiety was measured using the Generalized Anxiety Disorder Assessment (GAD-7 [57]). This is a self-report questionnaire consisting of 7 items evaluated on a 4-point Likert scale (each 0–3: not at all, several days, more than half the days, nearly every day) and is used as a screening tool and measure of the severity of generalized anxiety. It differentiates between generalized anxiety disorder and often comorbid depression as two separate dimensions. The sum of the results range from 0 to 21. The results from 0 to 5 indicate a mild level of anxiety, from 6 to 10 a moderate level of anxiety, and from 11 to 15 a strong level of anxiety. This questionnaire may be particularly useful for assessing the severity of symptoms and controlling their change over time. In order to facilitate the assessment of these changes, the 7 questions of GAD relate to recent symptoms, e.g., in the last 2 weeks. The Cronbach α coefficient of reliability was calculated as 0.92, with intraclass correlation calculated as 0.83 [57]. Further research also showed that GAD-7 is a reliable and valid measure to capture anxiety symptoms [58,59]. To our best knowledge, there has been no study undertaken in order to evaluate the psychometric properties of GAD-7 based on the Polish cohort; thus, we followed the norms proposed by Spitzer et al. [57]. For the current study, the Cronbach’s alpha reliability coefficient was 0.92 for the full scale. 

#### 2.2.4. Maternal Stress

Maternal stress was measured using the Parental Stress Scale (PSS [60]; Polish translation for Bieleninik). This is a self-report questionnaire consisting of 18 items evaluated on a 5-point Likert scale (strongly disagree, disagree, undecided, agree, and strongly agree) used to assess the stress level associated with parenthood. The sum of the results range from 18 to 90, with high scores indicating higher levels of stress. The questionnaire considers both positive (e.g., emotional benefits and personal development) and negative (demands on resources and restrictions) aspects of parenting. The Cronbach α coefficient of reliability was calculated as 0.83 with the inter-item correlation as 0.23 [60]. The Cronbach α coefficient of reliability in this study group was 0.84 for the full scale. 

#### 2.2.5. Infant’s Characteristics

The following medical items of children were collected from hospital records: child gender (male/female), birth weight (grams), delivery route (vaginal/cesarian), and final APGAR score (APGAR points: A = appearance (skin color), P = pulse (heart rate), G = grimace (reflex irritability), A= activity (muscle tone), and R = respiration).

#### 2.2.6. Socio-Demographic Information

The following socio-demographic information was collected from mothers:

- age (years);
- marital status (single, married, living together, not married, divorced, separated, widowed, and other);
- education level (primary/elementary or less; secondary school but not completed; secondary school graduate; university/college but not completed; and university degree (bachelor, master, PhD, or equivalent));
- work situation (employed full-time; employed part-time; self-employed; student full-time or part-time; stay-at-home parent; and unemployed and/or seeking work); and
- number of children in the household including the newborn participating in this study.

### 2.3. Statistical Analysis

Data analysis was performed by the SPSS program, version 26. Descriptive statistics (mean (SD); n (%)) were generated for all the variables included in this study.

1. In the first step, we calculated the Pearson correlation coefficient in order to verify the relationship between PBQ and the maternal variables (postnatal depressive symptoms, anxiety, and stress), and Spearman’s correlation coefficient for socio-demographic characteristics and the child’s characteristics variables.
2. The second set of analyses included examining the predictors of maternal postpartum bonding; thus, we calculated the linear regression between postpartum bonding and those variables, which were statistically significant in the first phase of the correlation analysis.
3. To test the mediating models, we followed Model 4 of the PROCESS macro for SPSS [61]. We used 5000 bootstrap resamples to generate CIs for the indirect effect of maternal postpartum depression on maternal postpartum bonding via maternal stress and for the indirect effect of maternal anxiety on maternal postpartum bonding via maternal stress.
4. Finally, we undertook a moderator analysis following Model 1 of the PROCESS macro for SPSS [61]. This analysis included maternal postpartum bonding and those variables that were statistically significant were also statistically significant in the first phase.

## 3. Results

### 3.1. The Characteristics of the Study Group

Eligible participants who matched the selection criteria were recruited between April 2019–March 2021. Of the 100 women included in the project, 72 fully completed the PBQ. The mothers’ average age was M = 31.44 years old (SD = 4.81). Table 1 shows the socio-demographic characteristics of the sample. The majority of participants were in a relationship (80.6% were married and 16.7% in partnership) and 70.8% declared to be employed full-time. Furthermore, a majority of included woman had a university degree (56.9% masters and 16.7% bachelors or equivalent), followed by those who completed secondary school (18.1%). In terms of the number of children in the family, 43.1% had two children, 29.2% had one child, and 18.1% had three children. 

All of the children were delivered before 37 weeks of gestation with an average gestational age of M = 33.54 (SD = 2.62; range 24–36). In terms of the sub-categories of preterm birth with regard to cut-off weeks of gestational age at delivery, the majority of newborns were late preterm (34 to 36 weeks and 6 days; N = 47; 65.4%), followed by moderate preterm (32 to 33 weeks and 6 days; N = 15; 20.8%), very preterm (28 to 31 weeks and 6 days; N = 7; 9.7%), and extremely preterm (<28 weeks; N = 3; 4.1%). 

Among the children, 55.6% were girls and 63.9% were delivered by caesarean section with the average weight in grams of M = 2201.58 (SD = 602). The average result on the APGAR scale was M = 8.42 (SD = 1.45), while only 23.6% of children received the maximum number of points on the Apgar scale.

### 3.2. Maternal Postpartum Bonding Scores after Preterm Birth

The average level of postpartum bonding is M = 7.87 points (range 0–41; SD = 7.18), indicating a very good emotional relationship between the mother and the newborn child. As can be seen from Table 2, the lowest mean of PBQ was observed for those newborns will the highest gestational age (34 to <37 weeks), indicating a better outcome in PBQ.

### 3.3. Maternal Stress, Anxiety, and Depressive Symptoms Scores

Table 3 presents descriptive characteristics for maternal stress, anxiety, and depressive symptoms scores. The average result obtained in the GAD-7 questionnaire is M = 13.60 (range 7–28; SD = 5.35), indicating a moderate severity of anxiety symptoms in the examined women. Only 16 mothers (22.2%) experienced mild anxiety (cut off at 5) and 30 (41.6%) experienced moderate anxiety (cut off at 10), while 20 (27.7%) experienced strong anxiety (cut off at 15; missing data for N = 6; 8.5%). In the case of stress, the examined women showed a relatively low level (32.81 points; range 19–58; SD = 7.80). Maternal experience of depression symptoms based on the obtained scores was also very low (M = 8.83; SD = 4.73), as only 9 mothers (13.4%) had a score greater than 13, indicating postpartum depression.

### 3.4. Correlations between Maternal Postpartum Bonding and Study Variables

The analysis aimed to indicate the initial correlations between Maternal-Infant bonding and postnatal depressive symptoms, stress, and anxiety in the tested group. The obtained Pearson correlation results showed strong positive correlations between the overall result of maternal bonding and stress. The remaining correlations were not statistically significant (Table 4).

Further statistical tests revealed a lack of significant Spearman correlations between the child’s variables (gestational age, birth weight, and final APGAR score) and socio-demographical variables (age, education level, number of children in the household) with postpartum bonding (Table 5).

The obtained results show a positive, moderate correlation between Maternal-Infant bonding and both educational level and number of children. In addition, a positive but rather weak correlation appeared between Maternal-Infant bonding and maternal age. The remaining correlations were not statistically significant.

### 3.5. Predictors of Maternal Postpartum Bonding

The next stage of the research analysis focused on the relationship between the postpartum bonding and the research variable, limited to maternal stress and maternal age. Sociodemographic variables, child’s characteristics, and maternal depressive symptoms and anxiety were excluded from the regression analysis due to the lack of significant correlations between these variables and maternal postpartum bonding, as demonstrated in the first phase of the correlation analysis. Even the education level and number of children in the household reached statistical significancy in Spearman correlation; thus, we decided to exclude it from regression analysis due to too large diversity and considering the small sample size (Table 4 and Table 5).

Regression analysis was performed and Maternal-Infant bonding played the role of the explained variable, while stress represented the explanatory variable. The model we tested revealed to be well suited to data F (2,63) = 30.689, *p* < 0.01, and explained 49% of the variability of the dependent variable. Conducted regression analysis in the studied group showed the significant role of the overall scores of stress experience (measured with the PSS). It can be concluded that the higher the stress score in the studied group, the more problematic postpartum bonding is between a mother and her child. The results obtained are presented in the table below (Table 6).

### 3.6. Mediating Models 

Following the literature [62], we created two mediating models: a mediating model of postpartum depressive symptoms on maternal postpartum bonding via maternal stress and a mediating model of maternal anxiety on maternal postpartum bonding via maternal stress. We wanted to investigate whether depressive symptoms and anxiety are intermediate variables that show statistically significant relations between maternal postpartum bonding and maternal stress considering the control of socio-demographical variables (maternal age, education level, and number of children in the household). 

**Figure 1:**
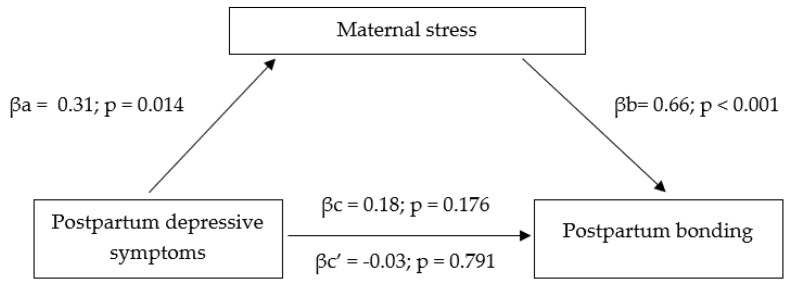
Mediating model of postpartum depressive symptoms on maternal postpartum bonding via maternal stress.

To test the first model, we used 5000 bootstrap resamples to generate CIs for the indirect effect of maternal postpartum depression on maternal postpartum bonding via maternal stress (effect = 0.21; Bootstrap lower level 95% CI (LLCI) = 0.04; Bootstrap upper level 95% CI (ULCI) = 0.39). The tested model (Figure 1) proved that the path from postpartum depression to maternal postpartum bonding (considering maternal stress in the model) is statistically insignificant (p = 0.791).

**Figure 2:**
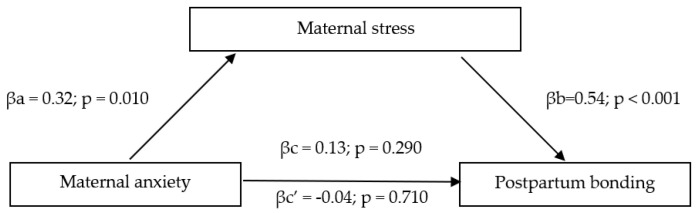
Mediating model of maternal anxiety on maternal postpartum bonding via maternal stress.

In terms of the second model, we used 5000 bootstrap resamples to generate CIs for the indirect effect of maternal anxiety on maternal postpartum bonding via maternal stress (effect = 0.17; Bootstrap lower level 95% CI (LLCI) = 0.04; Bootstrap upper level 95% CI (ULCI) = 0.34). The tested model (Figure 2) proved that the path from maternal anxiety to maternal postpartum bonding (considering maternal stress in the model) is statistically insignificant (p = 0.711). 

Overall, the mediating models revealed the significant affect of direct relationships between the analyzed variables in both cases. However, the relationship between postpartum depressive symptoms (the first model) and maternal anxiety (the second model) through stress on maternal postpartum bonding remained statistically insignificant.

### 3.7. Moderators of the Relationship between Maternal Postpartum Bonding and Maternal Stress

Finally, we undertook a moderator analysis to evaluate whether the relationship between maternal postpartum bonding and maternal stress depends on the value of those variables, which were statistically significant in the first phase of the analysis. Therefore, we considered socio-demographical variables as potential moderators (Table 7). The results showed no associations between the tested variables. This indicates that the relationship between maternal postpartum bonding and maternal stress is not moderated by the value of maternal age, education level, and number of children in the household. 

## 4. Discussion

Considering the importance of maternal postpartum bonding in regard to a child’s development and in the circumstance of premature birth, this study aimed to elucidate maternal postpartum bonding and its associations with maternal mental health, socio-demographical factors, and a child’s characteristics among mothers in the early postpartum period. The most relevant finding showed that the stress experienced by mothers was found to be a predictor of maternal postpartum bonding among the tested variables. Moreover, mothers of prematurely born children had a good level of Maternal-Infant bonding. The results’ analyses have shown positive correlations between the overall result of maternal postpartum bonding, stress, and socio-demographical variables (maternal age, education level, and the number of children in the household). Surprisingly, the study results did not show significant relationships between maternal postpartum bonding, maternal mental health (anxiety and depressive symptoms), and a child’s characteristics (gestational age, birth weight, and APGAR scale). In contrast to our expectations, maternal stress did not mediate the relationship between depression and maternal postpartum bonding, nor anxiety and maternal postpartum bonding. Furthermore, that relationship of maternal postpartum bonding and maternal stress was not moderated by the value of socio-demographical variables.

### 4.1. What Is the Maternal Postpartum Bonding Score among Mothers after Preterm Birth?

With respect to the first research question, the findings of the study indicated that mothers of prematurely born children had a good level of postpartum bonding with the infant (the average level of postpartum bonding was 7.87 points). 

Results from previous studies examining the impact of premature birth on parental bonding are inconsistent and often contradictory. Some studies showed that the process of bond development between a mother and her child can be disrupted by preterm birth [32]. In addition, a premature birth experience is associated with a more problematic bonding process with the newborn child [38,40]. However, other findings suggested that despite the negative factors and difficult emotions elicited by the PTB, most parents are fully involved in parental care from the very beginning [63]. Moreover, Borghini et al. [63] explained that parental involvement specifically may be stimulated by parental emotional states such as anxiety or worrying. A systematic review conducted by Korja et al. [64] indicated that mothers and their preterm-born babies are able to form as secure attachments as mothers of full-term babies. In their analysis, 5 out of 18 studies showed a similar or even higher quality of maternal interactions after premature labor in comparison to full-term birth. It is important to note that our study sample consisted of mothers for which almost two thirds of them experienced late preterm birth between 34 to <37 weeks (65.4%). Extremely preterm birth may be associated with a higher risk of parental fear about a child’s health and illness after the experience of having their baby admitted to a NICU and enduring prolonged hospitalization [65]. Thus, it can be assumed that a more disruptive bonding process may occur among mothers with lower gestational age. Hall and colleagues [36] indicated that mothers of premature babies presented even higher bonding feelings in comparison with mothers of full-term babies based on gestational age. This is in accordance with the results of Koria et al. [64] who observed that PTB experience may stimulate more parental engagement and a higher level of compensatory care while being with their infant. Although, it must be mentioned that in the current study, the analysis of the sub-categories of preterm birth based on gestational age showed that in each sub-category, there were mothers with more disrupted postpartum bonding.

### 4.2. What Are the Mothers’ Scores in Relation to Maternal Stress, Anxiety, and Depression Symptoms?

Interestingly in examining the second research question, results showed that the study sample had a moderate severity of anxiety symptoms (the mean GAD-7 scores was 13.60) and a relatively low level of experienced stress based on the obtained scores (the mean PSS scores was 32.81). The majority of the examined mothers also did not experience depression symptoms (the mean EPDS scores was 8.83); only 13.4% of mothers had a score greater than 13, which may indicate occurrence of serious depressive symptoms directly after delivery. The finding related to anxiety coincides with previous studies showing that preterm delivery adversely impacts mothers’ psychological state by increasing their level of maternal anxiety [35]. However, current research results do not support the previous studies which suggested that PTB affects the maternal psychological state [35] such as through an increase in depression symptoms [19] and higher stress in mothers after PTB [14,22]. 

### 4.3. Is Postpartum Bonding Associated with Maternal Mental Health (Depression Symptoms, Anxiety, and Stress)?

Preterm birth entails immanent difficulties and is acknowledged as a very stressful experience for parents [38]. Research shows that mothers who had a preterm labor experience have significantly higher levels of stress in comparison with mothers of full-term babies [14,22]. Furthermore, PTB is related to other factors that can increase parental stress; for example, separation from the newborn baby and prolonged hospitalization, which often causes additional parental stress and negatively impacts the sense of being a parent [25]. Hoffenkamp et al. [39] observed that PTB is connected with a higher level of parental concerns and negative experiences in the postpartum period. These include a heightened concern about the newborn’s health and difficulties during the process of parent–infant bonding due to challenges related to caring for an immature and fragile baby [34,39]. In terms to the third research question, we found that maternal postpartum bonding (overall PBQ result) is strongly and positively related to the maternal level of stress. In addition, the linear regression analysis, in which maternal postpartum bonding was the explained variable, showed that stress experienced by mothers predicts the level of maternal bonding. Based on the obtained results, it can be stated that higher levels of stress among mothers of preterm-born children is related to more problematic Maternal-Infant bonding. This finding is in accordance with other findings in which a negative connection between parenting stress and Maternal-Infant bonding was observed [47,66,67]. Contrary to our expectations, in this study, there was no significant relationship between maternal postpartum bonding and either anxiety or depressive symptoms. These results do not support previous research in which it was found that maternal mental health issues might have adverse impacts on Maternal-Infant bonding in the early postpartum period [35]. A systematic review conducted by Ticheman et al. [68] indicated that depressive symptoms had a negative relationship with postanal maternal bonding in the extensive majority of research studies. As the relationship between maternal bonding and depressive symptoms is more explored in the literature, there are mixed results in terms of anxiety. Among eight studies that investigated the association between postnatal bonding with anxiety, only one research study found a significant positive relationship between anxiety and bonding quality; the remaining studies showed either negative or no associations between anxiety and maternal postnatal bonding, in which correlations were weak or moderate [68].

Considering the transition to parenthood is a very stressful situation, we evaluated two mediating models to check if depression and anxiety (independently) mediate the relationship between maternal postpartum bonding via maternal stress. The motivation to create models stemmed from previous studies [62] in which general stress partially mediated the effects of anxiety upon depression. The results of the current study indicated that there was no relationship between anxiety or depression and maternal postpartum bonding when maternal stress was included as a mediator. The evidence for this relationship is inconclusive in the literature. Our results do not match those observed in earlier studies. For instance, Bieleninik et al. [46] observed that paternal stress mediates the relationship between paternal anxiety and paternal postpartum bonding. Our results are partially in agreement with those obtained by Nordahl et al. [69] who found no direct relationship between adult attachment style dimensions and bonding with the child when parental stress was included as a mediator. However, when examining each adult attachment domain with Maternal-Infant bonding, Nordahl et al. [69] found that the relationship between those two variables was mediated by parenting stress. This demonstrated that parenting stress is important to the quality of maternal bonding with a child. The differences in the results indicate the need for more research on maternal postpartum bonding and potential mediators.

### 4.4. Is Postpartum Bonding Associated with a Child’s Characteristics (Gestational Age, Birth Weight, and Final Apgar Score)?

With respect to the fourth research question, the obtained results revealed a lack of significant correlations between maternal postpartum bonding and a child’s characteristics such as gestational age, birth weight, and final APGAR score. In contrary to our obtained result, some research findings suggested that the severity of the health status of the infant [37] and the infant’s visible characteristics connected with prematurity are related to the Maternal-Infant bonding process [38]. According to the study conducted by Hoffenkamp et al. [39], the lower the gestational age of the child, the more negative the parental perception of the baby. Similarly, other results showed that children with low birth weights were seen by their parents as less attractive [40]. In the current study, more than 60% of children received between 8 and 10 points and 23.6% received the maximum number of points on the Apgar scale, whereas less than 20% had less than 8 points. In addition, the majority of mothers in this study gave birth between 34–37 weeks of pregnancy. Thus, the obtained study results in terms of a child’s characteristics may be related to children’s better health conditions. 

### 4.5. Is Maternal Postpartum Bonding Associated with Socio-Demographical Variables (Education Level, Age, and Number of Children in the Household)?

The fifth research question examined whether maternal postpartum bonding is associated with socio-demographical variables such as age, education level, and number of children in the household. Positive correlations were found between socio-demographical variables (educational level, maternal age, and number of children) and maternal postpartum bonding. These results are in line with other research. For instance, a systematic review conducted by Korja et al. [64] showed that in two studies, low socioeconomic status was a predictor of problems in the mother–infant relationship. A study conducted by Lehnig et al. [70] also underlined the appropriate socio-demographic factors for maternal postpartum bonding. They found that higher maternal educational level hinders the postpartum bonding process [70]. Results of other studies have confirmed that advanced maternal age (above 35) is related to more problematic postpartum bonding [29,71]. In terms of the number of children in the household, other findings also confirm our results regarding the fact that mothers who have more children experience a lower level of bonding with the newborn than first-time mothers in the early postpartum period [72].

In addition, we tested whether the relationship between maternal postpartum bonding and maternal stress is moderated by educational level, maternal age, and the number of children. The results have shown that this relationship does not depend on socio-demographical variables. To the best of our knowledge, we can say that the moderating role of socio-demographic variables with Maternal-Infant bonding and stress is not explored. According to the systematic review conducted by [68], none of the demographic variables were consistently related to Maternal-Infant bonding quality. In terms of reproduction experience including variables such as the number of children or number of previous pregnancies, it was demonstrated that women who had a second or third child had lower prenatal maternal bonding, although a majority of reported correlations were weak [68].

### 4.6. Implication for Clinical Practice

As preterm birth is a global problem, various authors provide important insight to the ongoing debate about the mother–infant interactions following preterm labor. This study showed that maternal stress is associated with maternal postpartum bonding in mothers after PBT. Clinically, this is most relevant result that emphasizes the need of providing psychological support and resources for mothers who experience high levels of stress shortly after PTB in order to reduce their stress level. First moments of the postpartum period are crucial for the development of early parental bonding with an infant [73], especially after PTB which is considered a vulnerable circumstance due to many risk factors related to a child’s health. Health care professionals should support mothers not only to reduce their stress level but also inform them about existing resources to help with PTB challenges. Given the fact that PTB may be a traumatic experience and be related to difficult consequences, routine assessment of stress level and mental health status in the early postpartum period is of a key importance in order to identify women at risk of having bonding difficulties.

### 4.7. Implication for Further Research

Inconsistent findings among previous studies emphasize the need for further investigations of Maternal-Infant bonding after PTB in the early postpartum period. Developing more extensive theoretical models with mediators and moderators in order to obtain a more detailed picture of the relationships between maternal bonding and the factors that may impact the process of postnatal bond development would be beneficial. It would be also beneficial to examine the development of Maternal-Infant bonding in a prospective study to assess the trajectory of bonding. There are still many unanswered questions about the maternal postpartum bonding score among mothers in terms of the sub-categories of PTB with regard to cut-offs of gestational age. Additionally, further examination of the relationship between maternal mental health and socio-demographic factors with postpartum bonding is needed as the early parental relationship with a prematurely born infant may be a complex phenomenon.

### 4.8. Strengths 

The major strength of this study is the consideration of various variables (maternal mental state, a child’s characteristics, and socio-demographical factors) in relation to Maternal-Infant postpartum bonding. It provides a more comprehensive picture of factors that may or may not be related to the postpartum experience of bonding among mothers after PTB. A key strength of the present study was the dataset used. We believe that the population of mothers of pre-term babies included in the current study could be considered as representative of mothers after PTB at the national level. The mothers average age in the current study was 31.44 years of age, which is consisted with the age of mothers giving birth to children in Poland (the median age was 30). The majority of mothers in the current study were in a relationship (80.6% were married and 16.7% in partnership). This is also in line with legitimate and illegitimate births in Poland, indicating that the vast majority of births take place in legally contracted marriages. The majority of mothers in this study had a university degree (56.9% masters and 16.7% bachelor or equivalent), which corresponds with the data observed by Statistics Poland which shows that more than half of the women (52%) giving birth in Poland have higher education [74]. In addition, the distribution of children included the current study in each group of sub-categories of PTB with regard to cut-offs of gestational age is consistent with the trend for PTB in Poland. From the total number of live PTBs in Poland in 2019 (N = 27,926), the number of births divided by period of gestation of new born neonates showed that 0.2% of neonates were born below 22 weeks of gestation, 5.7% below 28 weeks, 9.6% between 28 and 31 weeks, and 84.5% between 32 and 36 weeks [75]. Another strength of this study is the collection of data in the early postpartum period (1–3 days post-partum), which is not observed in many previous studies. 

### 4.9. Limitations

In this investigation, there are several sources of limitations. First, the generalizability of the study findings is limited due to the small sample size and the fact that the study was conducted within one medical unit; thus, the obtained data should be interpreted cautiously. The establishment of a tie between a mother and child in the early postpartum period is a complex phenomenon [76]. In bonding theory, skin-to-skin contact serves mostly to enhance parental feelings, thoughts, and behaviors towards the baby. Wittkowski et al. [77] have reported that many parent-report assessment measures exist to evaluate the bonding between mother and child during the early post-partum period but in the case of PTB, it is challenging to assess the parent–infant interaction. These difficulties exist because preterm neonates are often separated from the caregivers, located in incubators, and hospitalized at the NICUs. In the current study, some mothers reported that singular items of PBQ were not relevant in their situation due to having no direct contact with their babies (e.g., item 8 “I love my baby to bits”; item 11 “I enjoy playing with my baby”). Even though the PBQ is one of the most frequently used self-reported questionnaires to measure bonding [78], using self-reports is connected with various biases that may affect the results of the study. In addition, the used methods (PBQ and PSS) may require further validation reports. In order to make proper use of this study, the findings should also be read with caution due to the included population. The study sample consisted of mothers for which more than almost two thirds of the labors were terminated with late preterm births between 34 to <37 weeks (65.4%). Therefore, these findings cannot be extrapolated to all mothers after PTB. The next point is that this study is limited to the maternal experience; however, examining paternal experiences after preterm birth would be very valuable. Furthermore, this study is unable to encompass the entire spectrum of parent–infant bonding in the early postpartum period. It was beyond the scope of this study to examine the group of mothers after full-term birth; however, a lack of comparison group is a serious limitation in this study. Being limited to mothers of preterm babies, this study lacks showing the impact of PTB on mother–infant bonding and rather explores the maternal postpartum bonding and its relationship with a mother’s mental health, socio-demographical factors, and a child’s characteristics immediately after premature birth. Overall, the reader should bear in mind that the present study is based on the cross-sectional study design that precludes causal conclusions. 

## 5. Conclusions

The findings in this study emphasize that the experience of stress and socio-demographical variables (maternal age, maternal education level, and number of children in the household) may impact the process of developing a Maternal-Infant bond among mothers after the preterm birth. The current findings add to a growing body of literature on maternal postpartum bonding evaluated immediately after PTB and highlight the need for interventions targeting those mothers who experience high levels of stress. Mother–infant dyads should be supported after PTB as the postpartum period can be highly sensitive for the growth of the mother–infant bond. In addition, in this study, particular attention has been given to the level of maternal postpartum bonding that was not disrupted by the experience of the preterm birth. Many studies suggested that prematurity does not generate a higher risk for insecure attachment when the infant reaches 12 months [70]. However, the findings are inconsistent. The key is to provide supportive resources at NICUs and hospitals for parents who struggle with stress and mental health states related to childbirth to enhance positive bonding development, which later facilitates the infant’s socio-emotional, behavioral, and cognitive growth.

## Figures and Tables

**Table 1:** Socio-demographic characteristics of the study sample.

Demographic Variable	Mothers	
	n	%
Marital status		
Single	0	0
Married	58	80.6
Partnership	12	16.7
Divorced	1	1.4
Separated	0	0
Widowed	0	0
Other	0	0
Missing	1	1.4
Work situation		
Employed full-time	51	70.8
Employed part-time	2	2.8
Self-employed	7	9.7
Student full-time	0	0
Student part-time	1	1.4
Stay-at-home parent	5	6.9
Unemployed and seeking work	0	0
Missing	6	8.2
Education level		
Primary/elementary or less	1	1.4
Secondary school but not completed	2	2.8
Secondary school graduate	13	18.1
University/college but not completed	0	0
University degree (Bachelors or equivalent)	12	16.7
University degree (Masters or equivalent)	41	56.9
University degree (PhD or equivalent)	2	2.8
Missing	1	1.4
Number of children in the family		
One	21	29.2
Two	31	43.1
Three	13	18.1
Four	3	4.2
Five	2	2.8
Six	1	1.4
Missing	1	1.4
Type of birth		
Vaginal birth	26	36.1
Caesaren birth	46	63.9
Sex of the child		
Boy	32	44.4
Girl	40	55.6
APGAR		
5	3	4.2
6	4	5.6
7	6	8.3
8	15	20.8
9	14	19.4
10	17	23.6
Missing	13	18.1

**Table 2:** Mean results of PBQ in terms of the sub-categories of preterm birth with regard to the cut-offs of gestational age.

PBQ	N	M	SD	Minimum	Maximum
Extremely preterm (<28 weeks)	3	7.33	3.21	5	11
Very preterm (28 to 31 weeks and 6 days)	7	9.00	8.26	1	26
Moderate preterm (32 to 33 weeks and 6 days)	15	8.00	6.52	1	21
Late preterm (34 to 36 weeks and 6 days)	47	7.70	7.55	0	41

Annotation: SD, standard deviation; M, mean.

**Table 3:** Descriptive characteristics of PBQ, EDPS, GAD, and PSS.

Variable	N	M	SD	Minimum	Maximum
Postpartum Bonding (PBQ)	72	7.87	7.18	0	41
Depressive Symptoms (EPDS)	67	8.83	4.73	1	21
Anxiety (GAD-7)	66	13.60	5.35	7	28
Parental Stress (PSS)	66	32.81	7.80	19	58

Annotation: SD, standard deviation; M, mean.

**Table 4:** Pearson correlation results of Maternal-Infant bonding with depressive symptoms, anxiety, and stress.

	EPDS	GAD	PSS
PBQ average scores	0.227	0.168	0.696 **

** *p* < 0.01. Annotation: PBQ, Postpartum Bonding Questionnaire; EPDS, Edinburgh Postnatal Depression Scale; GAD-7, Generalized Anxiety Disorder Assessment; and PSS, Parental Stress Scale.

**Table 5:** Spearman correlation results of Maternal-Infant bonding with socio-demographic variables and child’s characteristics.

Variable	PBQ
Gestational age	−0.043
Birth weight	−0.055
APGAR scale	0.052
Maternal age	0.244 *
Educational level	0.386 **
Number of children	0.317 **

** *p* < 0.01, * *p* < 0.05. Annotation: PBQ, Postpartum Bonding Questionnaire.

**Table 6:** Results of the regression analyses for maternal postpartum bonding.

Predictor	β	B	SE	*p*	t
Parental Stress (PSS)	0.667	0.633	0.086	0.001	7.402
Maternal age	0.096	0.147	0.140	0.298	1.050
Constant value		−17.493	4.782	0.001	−3.658

R = 0.70 and R^2^ = 0.49. Annotation: β, the standardized beta; B, unstandardized beta; SE, standard error; *p*, significant; and t, the test statistic.

**Table 7:** Regression analysis of socio-demographical variables on the relationship between maternal stress and maternal postpartum bonding.

Predictor × Moderator	Dependent Variable	β	SE	t	*p*	LL	UL
Maternal stress × maternal age		0.032	0.022	1.47	0.14	−0.011	0.077
Maternal stress × educational level	postpartum bonding	0.068	0.077	0.89	0.37	−0.085	0.222
Maternal stress × number of children		−0.014	0.062	−0.202	0.84	−0.152	0.124

Annotation: β, the standardized beta; SE, standard error; *p*, significant; t, the test statistic; LLCI, lower level 95% CI; and ULCI, upper level 95% CI.

## Data Availability

The data that support the findings of this study are available on request from the corresponding author. The data are not publicly available due to their containing information that could compromise the privacy of research participants.

## References

[1] WHO (1977). Recommended definitions, terminology and format for statistical tables related to the perinatal period and use of a new certificate for cause of perinatal deaths. Modifications recommended by FIGO as amended 14 October 1976. Acta Obstet. Gynecol. Scand..

[2] Goldenberg R.L., Culhane J.F., Iams J.D., Romero R. (2008). Epidemiology and causes of preterm birth. Lancet.

[3] Blencowe H., Cousens S., Chou D., Oestergaard M., Say L., Moller A.-B., Kinney M., Lawn J. (2013). The born too soon preterm birth action group. The global epidemiology of 15 million preterm births. Reprod. Health.

[4] Chawanpaiboon S., Vogel J.P., Moller A.-B., Lumbiganon P., Petzold M., Hogan D., Landoulsi S., Jampathong N., Kongwattanakul K., Laopaiboon M. (2018). Global, regional, and national estimates of levels of preterm birth in 2014: A systematic review and modelling analysis. Lancet Glob. Health.

[5] Chang H.H., Larson J., Blencowe H., Spong C.Y., Howson C.P., Cairns-Smith S., Lackritz E.M., Lee S.K., Mason E., Serazin A.C. (2012). Preventing preterm births: Analysis of trends and potential reductions with interventions in 39 countries with very high human development index. Lancet.

[6] Liu L., Johnson H.L., Cousens S., Perin J., Scott S., Lawn J., Rudan I., Campbell H., Cibulskis R., Li M. (2012). Global, regional, and national causes of child mortality: An updated systematic analysis for 2010 with time trends since 2000. Lancet.

[7] Saigal S., Doyle L. (2008). Preterm birth 3. An overview of mortality and sequelae of preterm birth form infancy to adulthood. Lancet.

[8] Underwood M., Danielsen B., Gilbert W.M. (2007). Cost, causes and rates of rehospitalization of preterm infants. J. Perinatol..

[9] Manuck T.A., Rice M.M., Bailit J.L., Grobman W.A., Reddy U.M., Wapner R., Thorp J.M., Caritis S.N., Prasad M., Tita A. (2016). Preterm neonatal morbidity and mortality by gestational age: A contemporary cohort. Am. J. Obstet. Gynecol..

[10] Synnes A., Hicks M. (2018). Neurodevelopmental outcomes of preterm children at school age and beyond. Clin. Perinatol..

[11] Vohr B.R. (2014). Neurodevelopmental outcomes of extremely preterm infants. Clin. Perinatol..

[12] Vogel J.P., Chawanpaiboon S., Moller A.-B., Watananirun K., Bonet M., Lumbiganon P. (2018). The global epidemiology of preterm birth. Best Pr. Res. Clin. Obstet. Gynaecol..

[13] Carter J.D., Mulder R.T., Darlow B.A. (2007). Parental stress in the NICU: The influence of personality, psychological, pregnancy and family factors. Pers. Ment. Health.

[14] Ionio C., Colombo C., Brazzoduro V., Mascheroni E., Confalonieri E., Castoldi F., Lista G. (2016). Mothers and fathers in NICU: The impact of preterm birth on parental distress. Eur. J. Psychol..

[15] Jotzo M., Poets C.F. (2005). Helping parents cope with the trauma of premature birth: An evaluation of a trauma-preventive psychological intervention. Pediatrics.

[16] Cook N., Ayers S., Horsch A. (2018). Maternal posttraumatic stress disorder during the perinatal period and child outcomes: A systematic review. J. Affect. Disord..

[17] Brunson E., Thierry A., Ligier F., Vulliez-Coady L., Novo A., Rolland A.-C., Eutrope J. (2021). Prevalences and predictive factors of maternal trauma through 18 months after premature birth: A longitudinal, observational and descriptive study. PLoS ONE.

[18] Holditch-Davis D., Bartlett T.R., Blickman A.L., Miles M.S. (2003). Posttraumatic stress symptoms in mothers of premature infants. J. Obstet. Gynecol. Neonatal Nurs..

[19] Eduardo J.A.F.D.P., de Rezende M.G., Menezes P., Del-Ben C.M. (2019). Preterm birth as a risk factor for postpartum depression: A systematic review and meta-analysis. J. Affect. Disord..

[20] Grekin R., O’Hara M.W. (2014). Prevalence and risk factors of postpartum posttraumatic stress disorder: A meta-analysis. Clin. Psychol. Rev..

[21] Vigod S.N., Villegas L., Dennis C.-L., E Ross L. (2010). Prevalence and risk factors for postpartum depression among women with preterm and low-birth-weight infants: A systematic review. BJOG Int. J. Obstet. Gynaecol..

[22] Singer L.T., Salvator A., Guo S., Collin M., Lilien L., Baley J. (1999). Maternal psychological distress and parenting stress after the birth of a very low-birth-weight infant. JAMA.

[23] Alkozei A., McMahon E., Lahav A. (2014). Stress levels and depressive symptoms in NICU mothers in the early postpartum period. J. Matern. Neonatal Med..

[24] Abidin R.R. (1992). The determinants of parenting behavior. J. Clin. Child Psychol..

[25] Flacking R., Lehtonen L., Thomson G., Axelin A., Ahlqvist S., Moran V.H., Ewald U., Dykes F. (2012). The SCENE group closeness and separation in neonatal intensive care. Acta Paediatr..

[26] Bieleninik Ł. (2012). Dzieci Urodzone Przedwcześnie w Percepcji Matek (Mothers’ Perception of Prematurely Born Children in the Period of the Early Childhood).

[27] Gondwe K.W., Brandon D., Yang Q., Malcom W.F., Small M.J., Holditch-Davis D. (2019). Emotional distress in mothers of early-preterm infants, late-preterm infants, and full-term infants in Malawi. Nurs. Outlook.

[28] Demier R.L., Hynan M.T., Hatfield R.F., Varner M., Harris H.B., Manniello R.L. (1999). A measurement model of perinatal stressors: Identifying risk for postnatal emotional distress in mothers of high-risk infants. J. Clin. Psychol..

[29] Kinsey C.B., Hupcey J.E. (2013). State of the science of Maternal-Infant bonding: A principle-based concept analysis. Midwifery.

[30] Nakano M., Upadhyaya S., Chudal R., Skokauskas N., Luntamo T., Sourander A., Kaneko H. (2019). Risk factors for impaired maternal bonding when infants are 3 months old: A longitudinal population based study from Japan. BMC Psychiatry.

[31] Chandra P.S., Desai G., Reddy D., Thippeswamy H., Saraf G. (2015). The establishment of a mother-baby inpatient psychiatry unit in India: Adaptation of a Western model to meet local cultural and resource needs. Indian J. Psychiatry.

[32] Kantrowitz-Gordon I. (2013). Expanded care for women and families after preterm birth. J. Midwifery Women’s Health.

[33] Aagaard H., Hall E.O. (2008). Mothers’ experiences of having a preterm infant in the neonatal care unit: A meta-synthesis. J. Pediatric Nurs..

[34] Trumello C., Candelori C., Cofini M., Cimino S., Cerniglia L., Paciello M., Babore A. (2018). Mothers’ depression, anxiety, and mental representations after preterm birth: A study during the infant’s hospitalization in a Neonatal Intensive Care Unit. Front. Public Health.

[35] Khemakhem R., Bourgou S., Selmi I., Azzabi O., Belhadj A., Siala N. (2020). Preterm birth, mother psychological state and mother infant bonding. Tunis. Med..

[36] Hall R.A.S., Hoffenkamp H.N., Tooten A., Braeken J., Vingerhoets A.J.J.M., Van Bakel H.J.A. (2014). Child-rearing history and emotional bonding in parents of preterm and full-term infants. J. Child Fam. Stud..

[37] Soltis J. (2004). The signal functions of early infant crying. Behav. Brain Sci..

[38] Müller-Nix C., Ansermet F., Zeanah C.H. (2009). Prematurity, risk and protective factors. Handbook of Infant Mental Health.

[39] Hoffenkamp H.N., Tooten A., Hall R.A., Croon M.A., Braeken J., Winkel F.W., Vingerhoets A.J., van Bakel H.J. (2012). The impact of premature childbirth on parental bonding. Evol. Psychol..

[40] Goldberg S., DiVitto B., Bornstein M.H. (2002). Parenting children born preterm. Handbook of Parenting: Children and Parenting.

[41] Duley L., Uhm S., Oliver S. (2014). Top 15 UK research priorities for preterm birth. Lancet.

[42] Agnafors S., Bladh M., Svedin C.G., Sydsjö G. (2019). Mental health in young mothers, single mothers and their children. BMC Psychiatry.

[43] Sauer M.V. (2015). Reproduction at an advanced maternal age and maternal health. Fertil. Steril..

[44] Heath D.T., Mckenry P.C., Leigh G.K. (1995). The consequences of adolescent parenthood on men’s depression, parental satisfaction, and fertility in adulthood. J. Soc. Serv. Res..

[45] Hodgkinson S., Beers L., Southammakosane C., Lewin A. (2013). Addressing the mental health needs of pregnant and parenting adolescents. Pediatrics.

[46] Bieleninik Ł., Lutkiewicz K., Jurek P., Bidzan M. (2021). Paternal postpartum bonding and its predictors in the early postpartum period: Cross-sectional study in a polish cohort. Front. Psychol..

[47] Lutkiewicz K., Bieleninik Ł., Cieślak M., Bidzan M. (2020). Maternal-infant bonding and its relationships with maternal depressive symptoms, stress and anxiety in the early postpartum period in a Polish sample. Int. J. Environ. Res. Public Health.

[48] Brockington I.F., Oates J., George S., Turner D., Vostanis P., Sullivan M., Loh C., Murdoch C. (2001). A screening questionnaire for mother-infant bonding disorders. Arch. Women’s Ment. Health.

[49] Brockington I.F., Aucamp H.M., Fraser C. (2006). Severe disorders of the mother-infant relationship: Definitions and frequency. Arch. Women’s Ment. Health.

[50] Wittkowski A., Wieck A., Mann S. (2007). An evaluation of two bonding questionnaires: A comparison of the mother-to-infant bonding scale with the postpartum bonding questionnaire in a sample of primiparous mothers. Arch. Women’s Ment. Health.

[51] Wittkowski A., Williams J., Wieck A. (2010). An examination of the psychometric properties and factor structure of the post-partum bonding questionnaire in a clinical inpatient sample. Br. J. Clin. Psychol..

[52] Cox J.L., Holden J.M., Sagovsky R. (1987). Detection of postnatal depression. Development of the 10-item Edinburgh postnatal depression scale. Br. J. Psychiatry.

[53] Bielawska-Batorowicz E. (1995). Determinanty Spostrzegania Dziecka Przez Rodziców w Okresie Poporodowym.

[54] Marshall J., Bethell K. (2006). Edinburgh Postnatal Depression Scale (EPDS): Translated Versions.

[55] Kossakowska K. (2013). Edynburska Skala Depresji Poporodowej Właściwości Psychometryczne i Charakterystyka. Folia Psychol..

[56] Chrzan-Dętkoś M., Walczak-Kozłowska T. (2021). How do new mothers perceive screening for perinatal depression?. Health Psychol. Rep..

[57] Spitzer R.L., Kroenke K., Williams J.B.W., Löwe B. (2006). A brief measure for assessing generalized anxiety disorder. Arch. Intern. Med..

[58] Johnson S.U., Ulvenes P.G., Øktedalen T., Hoffart A. (2019). Psychometric properties of the general anxiety disorder 7-item (GAD-7) scale in a heterogeneous psychiatric sample. Front. Psychol..

[59] Löwe B., Decker O., Müller S., Brähler E., Schellberg D., Herzog W., Herzberg P.Y. (2008). Validation and standardization of the generalized anxiety disorder screener (GAD-7) in the general population. Med. Care.

[60] Berry J.O., Jones W.H. (1995). The parental stress scale: Initial psychometric evidence. J. Soc. Pers. Relatsh..

[61] Hayes A.F. (2017). Introduction to Mediation, Moderation, and Conditional Process Analysis: A Regression-Based Approach.

[62] Nima A.A., Rosenberg P., Archer T., Garcia D. (2013). Anxiety, affect, self-esteem, and stress: Mediation and moderation effects on depression. PLoS ONE.

[63] Borghini A., Pierrehumbert B., Miljkovitch R., Muller-Nix C., Forcada-Guex M., Ansermet F. (2006). Mother’s attachment representations of their premature infant at 6 and 18 months after birth. Infant Ment. Health J..

[64] Korja R., Latva R., Lehtonen L. (2012). The effects of preterm birth on mother-infant interaction and attachment during the infant’s first two years. Acta Obstet. Gynecol. Scand..

[65] Medina I.M.F., Granero-Molina J., Fernández-Sola C., Hernández-Padilla J.M., Avila M.C., Rodríguez M.D.M.L. (2018). Bonding in neonatal intensive care units: Experiences of extremely preterm infants’ mothers. Women Birth.

[66] De Cock E.S., Henrichs J., Klimstra T.A., Maas A.B., Vreeswijk C.M., Meeus W.H., van Bakel H.J. (2017). Longitudinal associations between parental bonding, parenting stress, and executive functioning in toddlerhood. J. Child Fam. Stud..

[67] Bailhache M., Doyle O., Salmi L., McDonnell T. (2019). Does maternal attachment to her infant mediate the link between perceptions of infant crying at 6 months and parenting stress at 24 months? A structural equation modelling approach. Child Care Health Dev..

[68] Tichelman E., Westerneng M., Witteveen A.B., Van Baar A.L., Van Der Horst H.E., De Jonge A., Berger M.Y., Schellevis F.G., Burger H., Peters L.L. (2019). Correlates of prenatal and postnatal mother-to-infant bonding quality: A systematic review. PLoS ONE.

[69] Nordahl D., Rognmo K., Bohne A., Landsem I.P., Moe V., Wang C.E.A., Høifødt R.S. (2020). Adult attachment style and maternal-infant bonding: The indirect path of parenting stress. BMC Psychol..

[70] Lehnig F., Nagl M., Stepan H., Wagner B., Kersting A. (2019). Associations of postpartum mother-infant bonding with maternal childhood maltreatment and postpartum mental health: A cross-sectional study. BMC Pregnancy Childbirth.

[71] Çalışır H., Karaçam Z. (2011). Factors associated with parenting behavior of mothers in the early postpartum period in Turkey. Nurs. Health Sci..

[72] Korucku O. (2018). Identification of factors affecting mother-infant bonding in advanced maternal age. Lupine Online J. Nurs. Health Care.

[73] Feldman R. (2015). Sensitive periods in human social development: New insights from research on oxytocin, synchrony, and high-risk parenting. Dev. Psychopathol..

[74] Statistics Poland (2020). Demographic Year Book of Poland.

[75] Statistics Poland. D.S.D. (2018). Demographic Situation of Poland up to 2017. Births and Fertility, 2018.

[76] Trombetta T., Giordano M., Santoniccolo F., Vismara L., Della Vedova A.M., Rollè L. (2021). Pre-natal attachment and parent-to-infant attachment: A systematic review. Front. Psychol..

[77] Wittkowski A., Vatter S., Muhinyi A., Garrett C., Henderson M. (2020). Measuring bonding or attachment in the parent-infant-relationship: A systematic review of parent-report assessment measures, their psychometric properties and clinical utility. Clin. Psychol. Rev..

[78] Perrelli J.G.A., Zambaldi C.F., Cantilino A., Sougey E.B. (2014). Instrumentos de Avaliação do Vínculo Entre Mãe e bebê.

